# cual-id: Globally Unique, Correctable, and Human-Friendly Sample Identifiers for Comparative Omics Studies

**DOI:** 10.1128/mSystems.00010-15

**Published:** 2015-12-22

**Authors:** John H. Chase, Evan Bolyen, Jai Ram Rideout, J. Gregory Caporaso

**Affiliations:** a Center for Microbial Genetics and Genomics, Northern Arizona University, Flagstaff, Arizona, USA; b Department of Biological Sciences, Northern Arizona University, Flagstaff, Arizona, USA; Dalhousie University

**Keywords:** bioinformatics, microbiome, metagenome, metabolome, transcriptome, genomes

## Abstract

The number of samples in high-throughput comparative “omics” studies is increasing rapidly due to declining experimental costs. To keep sample data and metadata manageable and to ensure the integrity of scientific results as the scale of these projects continues to increase, it is essential that we transition to better-designed sample identifiers. Ideally, sample identifiers should be globally unique across projects, project teams, and institutions; short (to facilitate manual transcription); correctable with respect to common types of transcription errors; opaque, meaning that they do not contain information about the samples; and compatible with existing standards. We present cual-id, a lightweight command line tool that creates, or mints, sample identifiers that meet these criteria without reliance on centralized infrastructure. cual-id allows users to assign universally unique identifiers, or UUIDs, that are globally unique to their samples. UUIDs are too long to be conveniently written on sampling materials, such as swabs or microcentrifuge tubes, however, so cual-id additionally generates human-friendly 4- to 12-character identifiers that map to their UUIDs and are unique within a project. By convention, we use “cual-id” to refer to the software, “CualID” to refer to the short, human-friendly identifiers, and “UUID” to refer to the globally unique identifiers. CualIDs are used by humans when they manually write or enter identifiers, while the longer UUIDs are used by computers to unambiguously reference a sample. Finally, cual-id optionally generates printable label sticker sheets containing Code 128 bar codes and CualIDs for labeling of sample collection and processing materials.

**IMPORTANCE** The adoption of identifiers that are globally unique, correctable, and easily handwritten or manually entered into a computer will be a major step forward for sample tracking in comparative omics studies. As the fields transition to more-centralized sample management, for example, across labs within an institution, across projects funded under a common program, or in systems designed to facilitate meta- and/or integrated analysis, sample identifiers generated with cual-id will not need to change; thus, costly and error-prone updating of data and metadata identifiers will be avoided. Further, using cual-id will ensure that transcription errors in sample identifiers do not require the discarding of otherwise-useful samples that may have been expensive to obtain. Finally, cual-id is simple to install and use and is free for all use. No centralized infrastructure is required to ensure global uniqueness, so it is feasible for any lab to get started using these identifiers within their existing infrastructure.

## Abstract (precis)

The adoption of identifiers that are globally unique, correctable, and easily handwritten or manually entered into a computer will be a major step forward for sample tracking in comparative omics studies. As the fields transition to more-centralized sample management, for example, across labs within an institution, across projects funded under a common program, or in systems designed to facilitate meta- and/or integrated analysis, sample identifiers generated with cual-id will not need to change; thus, costly and error-prone updating of data and metadata identifiers will be avoided. Further, using cual-id will ensure that transcription errors in sample identifiers do not require the discarding of otherwise-useful samples that may have been expensive to obtain. Finally, cual-id is simple to install and use and is free for all use. No centralized infrastructure is required to ensure global uniqueness, so it is feasible for any lab to get started using these identifiers within their existing infrastructure.

## INTRODUCTION

The number of samples in high-throughput comparative omics studies, such as those of microbiomes, metabolomes, genomes, and transcriptomes, is increasing rapidly due to the declining cost of experiments (e.g., DNA sequencing). This is often manifested in increasing sample replication, denser longitudinal or cross-sectional studies, or expansion of the exploration of a combinatorial experimental matrix (1–5). In this context, we define a sample as the finest-resolution unit being profiled, such as a single collection swab from which all 16S single-subunit rRNA will be sequenced in a microbiome survey.

To keep sample data and metadata manageable and to ensure the reliability of scientific results, we recommend using sample identifiers that are opaque (i.e., they should never contain sample metadata), correctable when transcription errors occur, and unique within an experiment (or, ideally, globally); they should also allow for the expansion of a data set without changing the labeling scheme and for integration with other projects without modification of the identifier (which simplifies metadata tracking). For these reasons, it is essential that we transition to well-designed sample identifiers.

In a typical microbiome study, sample identifiers are often defined on a per-study basis. For example, in a study tracking mouse gut microbial communities in treatment and control groups on a daily basis, the identifier format might be defined as follows: M<mouse number>.<timepoint>.

Whether each mouse received a treatment or the control is stored as metadata associated with this sample identifier, and this sample identifier is written on sample collection and processing materials (e.g., swabs or 50-ml conical tubes and subsequently microcentrifuge tubes of isolated DNA and amplicon pools). Specific identifiers adhering to this format might be M1.2015-10-21 or M3.2015-10-21, representing the samples taken from mouse 1 on 21 October 2015 and mouse 3 on 21 October 2015. While on the surface this seems to be a reasonable strategy for assigning identifiers (and similar to ones we have used in the past for our microbiome projects), it is problematic for several practical reasons.

First, sample identifiers containing metadata, such as date or subject identifiers, are prone to transcription errors that cannot be resolved. A single illegible character, such as a 3 that looks like an 8, when written in permanent marker on the side of a microcentrifuge tube can make it impossible to differentiate DNA from samples M3.2015-10-21 and M8.2015-10-21. In practice, this results in apparently having two samples of DNA for M8.2015-10-21 and none for M3.2015-10-21 and (in our experience) in throwing away data for both samples to avoid misleading results in the event of a bad guess about which DNA belongs to which sample.

Next, a change to the experimental design partway through the experiment can make the identifier format insufficient or obsolete. For example, if a decision is made to include technical replicates partway through an experiment, the identifier format would need to be adapted. In our experience, this would probably happen by appending R1, R2, etc. (for replicates 1 and 2) to the end of the identifiers. To make the identifier format consistent across all samples, this would require relabeling of already-collected samples. Alternatively, it would require that everyone involved (including people who may join the project at a later time) be aware of the different identifier formats and know how to interpret the meaning of the differences. While both of these are possible, it is preferable not to change or add sample identifier formats partway through a project.

Finally, in meta-analyses or projects where different groups collect samples that are centrally processed (such as the Earth Microbiome Project [3]), sample identifiers can easily conflict across individual projects if no effort is made up front to ensure global uniqueness. This often results in sample identifiers being reassigned, which then leads to confusion over sample provenance tracking when all parties who have worked with a set of samples in the past are not informed of the renaming. Further, the renaming will probably not be propagated through all sample metadata spreadsheets, field and lab notebooks, and other places where the identifiers were recorded, which leads to multiple identifiers that refer to the same sample.

To support the general-use case of sample identifiers in comparative omics and address the issues of transcription errors and obsoletion or insufficiency of sample identifiers, we propose that sample identifiers (i) be unique across projects and, ideally, globally across project teams; (ii) facilitate transcription by hand by being short and not containing visually ambiguous characters; (iii) be correctable with respect to common types of transcription errors; (iv) be opaque, meaning that they do not contain embedded metadata; (v) be compatible with metadata standards, such as the ISA-Tab (6) file format, and with minimal information requirements, such as those of the MIxS project (7); and (vi) ideally, support resolution so that identifiers can be mapped to sample metadata across project teams.

Uniqueness is a property that must be present in an identifier for it to be useful. In most cases, identifiers are defined to be unique within a given project, but the more globally unique an identifier can be, the more useful that identifier is. However, as the scope of the identifier increases, so does the complexity of managing its uniqueness. If an identifier is required only to be unique within a project, a single spreadsheet is likely sufficient to manage the identifiers and Unix command line tools (such as cut -f 1 | sort | uniq -d) are sufficient to identify violations. However, if an identifier should be globally unique, as with digital object identifiers (DOIs), infrastructure may need to be in place to support this, including a server(s) for assigning identifiers while ensuring uniqueness, a server(s) for resolving identifiers, and likely even a governing body that defines specifications, performs updates, and ensures sustainability.

Identifiers used in comparative omics studies should be as short as possible, as these are often written down (e.g., in field notebooks or on microcentrifuge tubes). This must be balanced with the fact that as identifiers become shorter, they are capable of encoding fewer samples, which is especially important when correctability is desired. For this same reason, identifiers should avoid using visually ambiguous characters such as 0 and O (zero and capital “oh”) or 1 and l (one and lowercase “el”).

Ideally, identifiers should not be subject to human transcription, though in practice this is often not feasible. Thus, identifiers should be correctable, such that if a transcription error occurs, it will be possible to determine (most importantly) that the identifier is wrong and (secondarily) what the correct identifier is. This is not possible for identifiers that are within an edit distance of one from each other (meaning that two identifiers are only one character different from each other), such as M3.2015-10-21 and M8.2015-10-21 from our example above. Common transcription error types, including substitution, transposition, omission, insertion, or duplication of characters, should be correctable to ensure that a reasonable amount of transcription error can be tolerated.

Sample identifiers should also be opaque, meaning that they do not encode information about the samples that they identify. Instead, that information should be associated with the identifier. This is a well-established principle in database management (for example, see chapter 8 of reference 8) but is counterintuitive for most biologists. While it seems useful to have an identifier represent information about the sample (we have done this for several of our own projects in the past), the initial naming scheme often needs revision partway through a project, as exemplified above. Further, these naming schemes usually (as a byproduct) are not correctable, because some valid identifiers have an edit distance of one from other identifiers.

Sample identifiers should be compatible with applicable metadata standards in their domain to ensure compatibility with existing tools. In comparative omics, the relevant standards of which we are aware include ISA-Tab, which is a file format specification describing in part how to represent sample metadata (where our identifiers align with names in ISA-Tab v1.0), and the MIxS specifications.

Finally, it is desirable for sample identifiers to be resolvable across projects. A resource where sample identifiers can be looked up to get all associated information about that sample would be extremely valuable. Similarly, a search resource where a user could query based on sample metadata (e.g., sample type, soil, a pH of 4.5 to 10.5, an Illumina-MiSeq sequencing platform) and get details on all samples with these metadata would be invaluable.

## RESULTS

**FIG 1:**
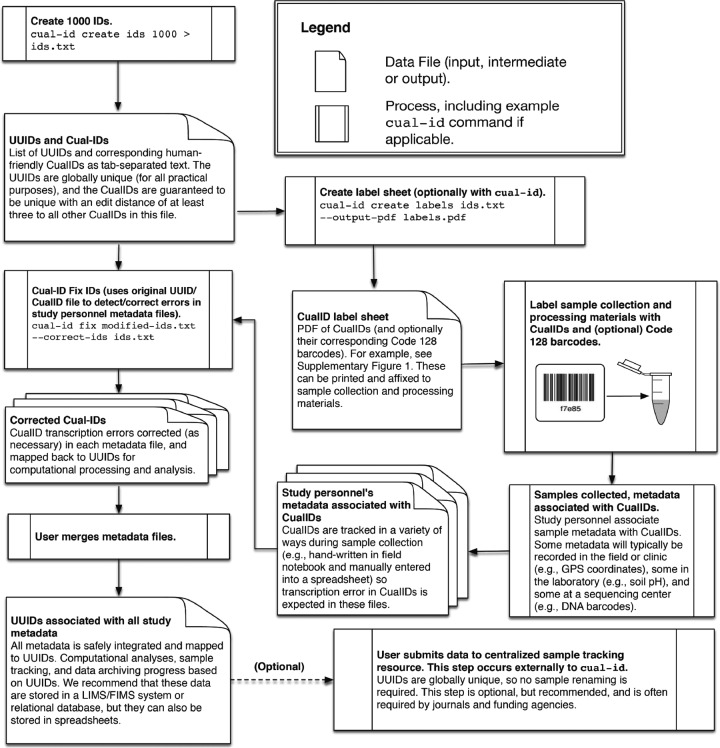
Diagram illustrating the intended use of cual-id.

We present cual-id (Fig. 1), a software package that creates, or mints, sample identifiers that meet criteria i to v above and around which resources can be built to support criterion vi. cual-id allows users to assign universally unique identifiers, or UUIDs (9), that are globally unique (for all practical purposes) to their samples. UUIDs are too long to be conveniently written on sampling materials (e.g., swabs, microcentrifuge tubes), so cual-id additionally generates human-friendly 4- to 12-character identifiers which we call CualIDs (distinguished by case from the name of the software package, which is called cual-id) that are guaranteed to be unique within a generated set of identifiers. UUIDs are intended to be used by computers, while CualIDs are intended to be used by humans when manual transcription is required. UUIDs map directly to CualIDs, and the mapping can be done by eye (they are the trailing characters of the full identifier) or programmatically with cual-id, which corrects transcription errors while mapping identifiers. Finally, cual-id allows users to generate PDFs of identifiers and (optionally) their corresponding Code 128 bar codes, formatted for printing on Electronic Imaging Materials CryoLabel sticker sheets (an example is provided in Fig. S1 in the supplemental material) to be used for labeling sampling materials.

10.1128/mSystems.00010-15.1 Figure S1 Example of cual-id PDF output. The Code 128 bar codes decode to the identifiers listed under each bar code (free smartphone apps are available for decoding bar codes and can be tested by scanning the bar codes in this PDF). This PDF is formatted for printing on Electronic Imaging Materials CryoLabel sticker sheets (catalog number 80402). Download Figure S1, PDF file, 0.01 MB. Copyright © 2015 Chase et al. 2015 Chase et al. This content is distributed under the terms of the Creative Commons Attribution 4.0 International license.

Other approaches exist for minting identifiers and ensuring uniqueness, including using UUID-assigning software directly and NOID (https://wiki.ucop.edu/display/Curation/NOID). cual-id provides convenient functionality above that of UUID-assigning tools and is useful for this problem domain (notably, it supports human transcription and error correction). NOID aims to support the creation of globally unique identifiers with error detection capabilities (e.g., credit card numbers) but uses a persistent infrastructure to support unique identifier generation, specifically a database. cual-id, as with UUID assignment generally, does not require a centralized service to assign globally unique identifiers.

cual-id is written in Python 3 and is available open source and free for all use under the BSD3 clause license. It is fully PEP 0008 compliant (https://www.python.org/dev/peps/pep-0008/). cual-id is accessible through command line and Python 3 application programming interfaces. Its command line interface is based on Click (http://click.pocoo.org/), and it uses ReportLab software for PDF generation. All source code, associated tests, and documentation are available on GitHub at https://github.com/johnchase/cual-id.

## DISCUSSION

Researchers involved in comparative omics studies generally work with their sample identifiers in spreadsheets, field notes, and lab notebooks and write them on sampling materials, such as collection swabs. While the need to manually transcribe and enter sample identifiers is likely to persist, ultimately, we hope that project teams will move away from management of sample data and metadata using spreadsheets and transition toward relational databases, field information management systems (FIMS) (see, e.g., reference 10), and laboratory information management systems (LIMS) (see, e.g., reference 11). These systems benefit users by offering features, including centralized sample tracking across projects; synchronized metadata between team members in the field, the wet lab, and the bioinformatics lab; and automated backups. Project teams within the biological sciences, such as the Moorea Biocode Project (http://mooreabiocode.org/ [11]), have begun using these systems in recent years, and as the size and scope of studies continue to grow, use of these systems will become increasingly important. However, we acknowledge that making this transition requires a considerable investment of time and money (though the latter two benefits can be attained by using online tools like Google Sheets, which we highly recommend in the interim for managing project spreadsheets). Because UUIDs are globally unique, they will directly support these approaches, as well as transitions toward centralized sample data/metadata tracking (e.g., across all labs at an institution or across all projects funded through a given program) as they come into more-widespread use, without reassigning identifiers. For example, UUIDs can be directly used as database primary keys by many existing database management systems, such as PostgreSQL, so spreadsheets of sample metadata generated now could trivially be transitioned into database tables. Use of cual-id will therefore move the field toward sustainable management of sample identifiers with very minimal investment up front.

We note that cual-id does not provide a resolution service for UUIDs. Rather, it provides users with a way to generate these for their own internal use and in a way that supports sharing of their data in systems that aim to integrate comparative omics data across project teams. Because the UUIDs generated with cual-id are globally unique, they are compatible with systems that integrate sample data across project teams to support centralized data storage and meta-analysis, such as Qiita (for microbiome data, http://qiita.ucsd.edu) or Integrated Microbial Genomes (IMG) Data Warehouse (for genome and metagenome data, http://img.jgi.doe.gov/). cual-id-generated UUIDs could be provided as sample identifiers now for these systems.

cual-id provides a framework for generating globally unique identifiers for biological samples that are easily transcribed by humans in the lab or in the field and are robust (within reason) to transcription errors. Using identifiers that are globally unique across studies and project teams but are also easily written on paper or manually entered into a spreadsheet will be a major step forward for quality control and sample tracking in comparative omics studies.

## MATERIALS AND METHODS

### UUID assignment.

cual-id mints a user-defined number of UUIDs for use as sample identifiers. This is performed with the cual-id create command. These identifiers are canonically represented with 32 hexadecimal characters (from the alphabet 0123456789abcdef) and are a commonly accepted form for primary keys in relational databases. cual-id creates version 4 UUIDs, which are randomly generated and contain 128 bits of information. The resulting identifiers are globally unique for practical purposes in that there are over 10^36^ of them (for comparison, it is estimated that there are about 10^29^ bacteria in Earth’s oceans [12]) so are widely used when globally unique identifiers need to be generated offline or in a decentralized manner.

### CualID assignment.

CualIDs are derived from full UUIDs as the last *n* characters of the UUID, where *n* is user defined and in the range of 4 to 12 (inclusive). These identifiers are intended to be transcribed by users, for example in field notebooks or in permanent marker on microcentrifuge tubes in the lab, so some transcription errors should be anticipated. CualIDs should therefore not contain visually ambiguous characters, and transcription errors should be correctable (within reason). Table 1 presents three cual-id-assigned UUIDs and their corresponding length 6 and length 8 CualIDs. The hexadecimal alphabet does not contain any pairs of visually ambiguous characters, so we use this alphabet to represent our CualIDs (which is commonly used for presenting UUIDs readable by humans).

**TABLE 1:** Sample UUIDs and their corresponding length 6 and length 8 CualIDs generated by cual-id

UUID	Length 6 CualID	Length 8 CualID
3cd7e2b8-70ea-41f1-ae99-fea5ff5ed2c4	5ed2c4	ff5ed2c4
24c715bc-b0e1-4808-b55f-e2645d4af925	4af925	5d4af925
3c094f4a-a1eb-4a78-bc74-c4e05b0434f6	0434f6	5b0434f6

**FIG 2:**
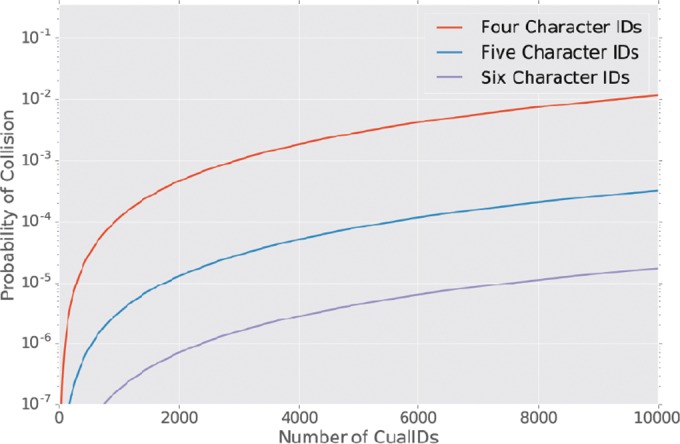
Probability of generating a duplicate CualID as a function of CualID length. Users can define their CualID length based on the number of samples in their study; more samples require longer CualIDs to support correctability.

Because CualIDs are designed to be short to facilitate human transcription, they are not globally unique, and the probability of generating two identical CualIDs is a function of the CualID length (*n*). For studies that need more CualIDs because they have more samples, longer CualIDs should be generated. Figure 2 illustrates the probability of getting a duplicate CualID as a function of CualID length (which is an example of the birthday problem). However, cual-id explicitly disallows the generation of CualIDs with an edit distance of less than three, to ensure that within a run of CualIDs the resulting identifiers will be correctable. For studies on the orders of 100, 1,000, 10,000, and 100,000 samples, we recommend CualIDs of lengths 4, 5, 6, and 8, respectively (but we note that generating 100,000 CualIDs can be very slow, as the process of computing edit distances between all CualIDs scales quadratically).

**FIG 3:**
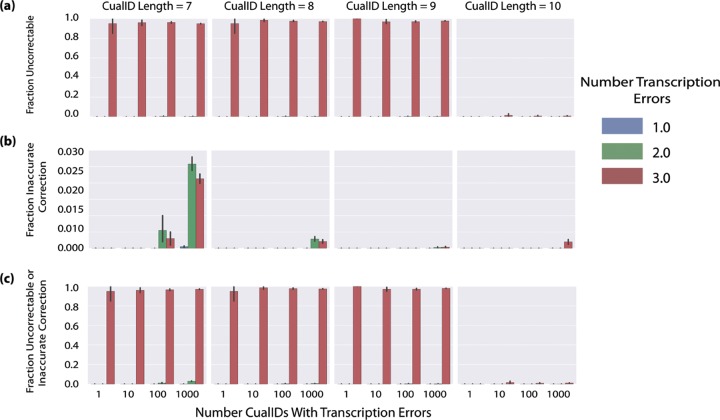
Frequency of errors in CualID correction as a function of CualID length (columns), numbers of CualIDs with transcription errors (*x* axis within each subplot), and numbers of errors introduced (colors). CualIDs were generated and errors were introduced randomly in each CualID identifier; each bar was computed based on 20 iterations. Error bars indicate standard deviations. (a) Fractions of false negatives, meaning that a CualID with transcription errors cannot be resolved, and no corrected CualID is returned. (b) Fractions of false positives, meaning that a CualID with transcription errors is incorrectly assigned to another identifier. Note that the scale on the *y* axis is much smaller in this panel than in panel a or c and indicates that the rate of false positivity is very low. (c) Fractions of either false positives or false negatives (i.e., the combination of values from panels a and b). The similarity of panels a and c illustrates that the number of false positives is negligible relative to that of false negatives.

To make CualIDs correctable, when minting identifiers, cual-id explicitly checks that the last *n* characters of the UUIDs (which become the CualIDs) that are minted have a Hamming distance (the number of positions at which two sequences of equal lengths differ) of at least two from all other UUIDs minted in that run of cual-id. This means that CualIDs minted within a run of cual-id are guaranteed to be correctable for small amounts of transcription error, such as the substitution of one or two characters. Figure 3 presents the results of a simulation to determine how common false-negative errors (a CualID with transcription errors cannot be assigned to its UUID) and false-positive errors (a CualID with transcription errors is assigned to the wrong UUID) occur based on the number of errors introduced, the number of CualIDs with errors, and the CualID length. This simulation shows that both types of error are extremely uncommon when fewer than three errors are present in the CualID, and when errors do occur, the vast majority are false negatives (which for this application are preferable to false positives).

While CualID transcription errors will also often be correctable across runs of cual-id, there is no guarantee placed on the edit distance in this case, as that begins to require more-complex infrastructure, such as a server, to support identifier minting. In practice, however, this should not be an issue, as the intended lifetime of the CualIDs is on the scale of a single project, which is the scale on which transcription errors generally need to be corrected (e.g., over one or a few DNA sequencing runs, and 10s of thousands of CualIDs can easily be generated in advance). When comparing data across projects, human transcription of sample identifiers should be complete, and the computationally stored and accessed UUIDs are not subject to transcription error.

### Error correction.

cual-id can correct common types of typographical errors in CualIDs, including substitution of one character for another, transposition of characters, omission of characters, insertion of characters, and duplication of characters. This can be performed using the cual-id fix command and is achieved by providing query CualIDs that need to be corrected (or are confirmed to be correct) and the list of known CualIDs (which will be generated by cual-id during the minting stage). Each query identifier is compared to all of the known identifiers, and the command puts out the corrected identifier for each query if possible, as well as any identifiers that were duplicated (which are therefore not resolvable). This is performed using Python’s difflib module, which compares sequences for similarity. The algorithm used is similar to Gestalt pattern matching, where sequences are matched based on finding subsequences that match between sequences first in the forward and then in the reverse direction. This is more useful for correcting identifiers than Hamming or Levenshtein distance, as it returns the identifier that a human would “expect.” For example, the identifiers 12345678 and 23456789 would not be identified as being similar by Hamming distance, but difflib would easily resolve them.
