# Supplementary figures and images for: cual-id: Globally Unique, Correctable, and Human-Friendly Sample Identifiers for Comparative Omics Studies
Source: mSystems. 2015 Dec 22;1(1):e00010-15. doi: 10.1128/mSystems.00010-15 (PMC5069752; doi:10.1128/mSystems.00010-15)

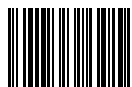

20cca

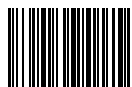

c1b01

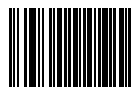

6b165

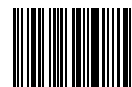

1de50

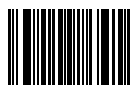

5128a

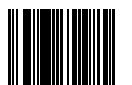

53047

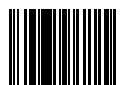

32034

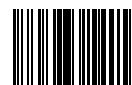

bb7c6

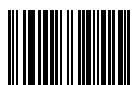

62eb5

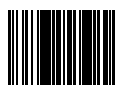

e2158

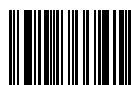

52eaf

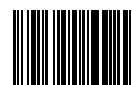

0f590

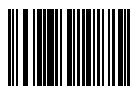

f7e85

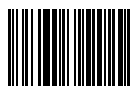

e69d0

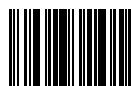

4c7b1

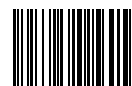

edf24

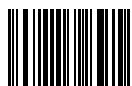

f21de

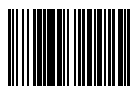

b76d2

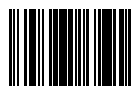

3b79f

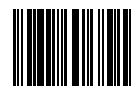

70a8f

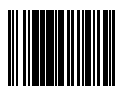

49200

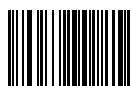

aed69

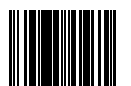

38746

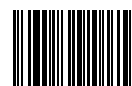

69b62

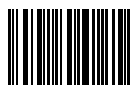

e8e98

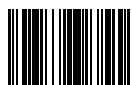

7ed7e

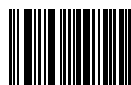

54c38

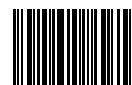

8989a

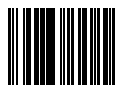

17831

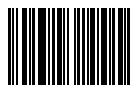

956c3

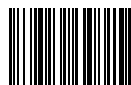

d9ca5

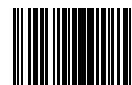

2f678

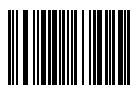

f19ad

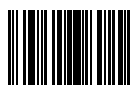

6a27e

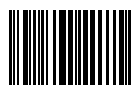

8e399

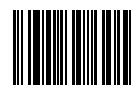

31e5a

Supplement: Figure S1 [file sys001160025sf1.pdf]
